# Evaluation of digital PCR for detecting low-level EGFR mutations in advanced lung adenocarcinoma patients: a cross-platform comparison study

**DOI:** 10.18632/oncotarget.18866

**Published:** 2017-06-29

**Authors:** Jincui Gu, Wanchun Zang, Bing Liu, Lei Li, Lixia Huang, Shaoli Li, Guanhua Rao, Yang Yu, Yanbin Zhou

**Affiliations:** ^1^ Department of Respiratory Medicine, The First Affiliated Hospital of Sun Yat-sen University, Guangzhou, China; ^2^ Novogene Bioinformatics Institute, Beijing, China

**Keywords:** EGFR, NSCLC, digital PCR, circulating tumor DNA

## Abstract

Emerging evidence has indicated that circulating tumor DNA (ctDNA) from plasma could be used to analyze EGFR mutation status for NSCLC patients; however, due to the low level of ctDNA in plasma, highly sensitive approaches are required to detect low frequency mutations. In addition, the cutoff for the mutation abundance that can be detected in tumor tissue but cannot be detected in matched ctDNA is still unknown. To assess a highly sensitive method, we evaluated the use of digital PCR in the detection of EGFR mutations in tumor tissue from 47 advanced lung adenocarcinoma patients through comparison with NGS and ARMS. We determined the degree of concordance between tumor tissue DNA and paired ctDNA and analyzed the mutation abundance relationship between them. Digital PCR and Proton had a high sensitivity (96.00% vs. 100%) compared with that of ARMS in the detection of mutations in tumor tissue. Digital PCR outperformed Proton in identifying more low abundance mutations. The ctDNA detection rate of digital PCR was 87.50% in paired tumor tissue with a mutation abundance above 5% and 7.59% in paired tumor tissue with a mutation abundance below 5%. When the DNA mutation abundance of tumor tissue was above 3.81%, it could identify mutations in paired ctDNA with a high sensitivity. Digital PCR will help identify alternative methods for detecting low abundance mutations in tumor tissue DNA and plasma ctDNA.

## INTRODUCTION

EGFR tyrosine kinase inhibitor (EGFR TKI) therapies have shown to be of great benefit to non-small cell lung cancer (NSCLC) patients harboring EGFR activating mutations, such as L858R and exon 19 deletions [1, 2]. However, most patients develop drug resistance one or two years after first-generation EGFR TKI treatment, and the T790M mutation accounts for over half of acquired resistance cases [3, 4, 5]. Since the third generation EGFI-TKI AZD9291 (Osimertinib) is highly effective in NSCLC patients with EGFR T790M mutation and has been approved by the US FDA [6, 7, 8], it is recommended that tumor tissue biopsy results be obtained to assess EGFR mutation status so that those patients can receive the appropriate treatment.

Tumor biopsy is usually used as the gold standard for detecting gene mutation. However, it might not supply a sufficient amount of tumor tissue for mutation analysis, and meanwhile, invasive intervention may be risky and discomforting for patients, especially in those who need serial biopsy to monitor treatment response or recurrence. Moreover, the gene mutations in a single tumor biopsy may not represent the overall gene mutation profiles of a patient with heterogeneous cancer or multiple metastases [9, 10]. Recently, circulating tumor DNA (ctDNA) has emerged as an alternative DNA source for detecting EGFR mutations. Several studies have demonstrated that the EGFR mutations detected in plasma ctDNA are highly concordant with those detected in the tumor tissue of patients [11, 12, 13], indicating that ctDNA in plasma can serve as an alternative to tissue biopsy. Although ctDNA has shown a great potential for clinical application, the amount of ctDNA appears to be very low, and the mutation abundance in ctDNA is lower than that in tumor tissue [14, 15, 16]. Therefore, a highly sensitive method with a low detection limit is required to provide precise and valuable mutation information for clinical decision-making [17].

Several methods for assessing EGFR mutation in NSCLC have been developed, such as the conventional Scorpion amplification refractory mutation system (ARMS), next-generation sequencing (NGS) and digital PCR. Both ARMS PCR and NGS have been widely used in clinical laboratories, but they have been challenged on the accuracy of detecting low mutation allele frequencies [18]. Digital PCR is a promising new technique that can provide an absolute quantification of gene mutations, without any external calibrators, and the greatest strength of digital PCR is its ability to identify mutation allele frequencies as low as 0.1%.

In the current study, we evaluated the outperformance of digital PCR in the identification of low frequency EGFR L858R, T790M and exon 19 deletion mutations in tumor tissue samples from advanced lung adenocarcinoma patients through comparison with those detected using Ion AmpliSeq Cancer Hotspot Panel V2 (Thermo Fisher Scientific, Waltham, MA) and ARMS PCR. To evaluate its performance in ctDNA mutation detection and analyze the relationship between the mutation abundances of tumor tissue and ctDNA, which might provide a clinical reference, we assessed the consistency between the mutation detection of tumor tissue and the mutation detection of ctDNA using the digital PCR platform and analyzed the cutoff value of tumor tissue mutation abundance that could detect mutation in the corresponding ctDNA.

## RESULTS

### Patient characteristics and sample collection

**Figure 1:**
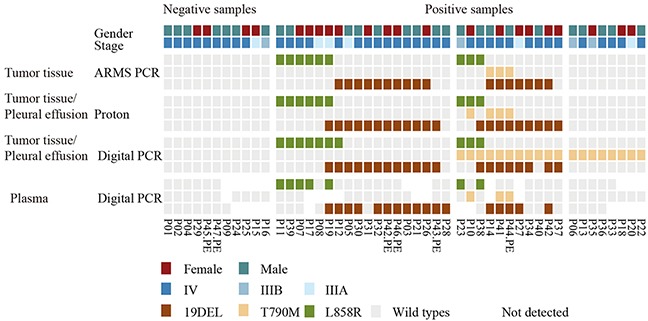
EGFR mutations detected by three platforms using tumor tissue and plasma samples A heatmap representation of mutations detected from paired tumor tissue/pleural effusion and plasma samples, which were collected from 47 patients. The sample types and platforms used are shown in the left column. Forty-seven tumor tissue samples were detected using ARMS PCR, while 42 tumor samples and 6 pleural effusion samples were detected using Proton and the digital PCR platform. Patients with tumor tissue samples in ARMS PCR, but with pleural effusion samples in Proton and digital PCR were marked with the symbol “PE”. P42 had both tumor tissue and pleural effusion samples detected in Proton and digital PCR. Forty-seven paired plasma samples were detected using digital PCR only. The top two rows show patients’ gender and clinical stage, the upper middle three rows show tumor tissue samples tested by ARMS PCR, the middle six rows show tumor tissue or pleural effusion samples tested by Proton and digital PCR, and the bottom three rows show plasma samples tested by digital PCR only. Mutation events are represented by different colors, e.g., L858R by green, T790M by yellow, 19del by brown, and wild-type mutations by gray. The sites of ctDNA that were not detected are shown as blank.

A total of forty-seven patients diagnosed with NSCLC adenocarcinoma were enrolled in this study. The patient cohort was composed of 21 females (44.68%) and 26 males (55.32%). The majority of patients were stage IV (36, 76.60%), with 6 (12.77%) patients being stage IIIB and 5 (10.64%) patients being stage IIIA. Forty-two patients were EGFR TKI treatment naive, while the other 5 patients experienced disease progression after first generation EGFR TKI treatment. The patient characteristics and sample types are presented in Table 1, and a full summary of patient characteristics and gene mutations in matched tumor tissue DNA and ctDNA detected by three platforms is presented in Figure 1 and Supplementary Table 1.

**Table 1:** Clinical features of 47 patients with non-small cell lung cancer

Characteristics	Number (%)
Sex	
Male	21 (44.68%)
Female	26 (55.32%)
Tumor stage	
IIIA	6 (12.77%)
IIIB	5 (10.64%)
IV	36 (76.60%)
EGFR-TKIs treatment history	
EGFR-TKIs naïve patients	42 (89.36%)
EGFR-TKIs experienced patients	5 (10.64%)
Sample type	
Tumor tissue	42 (89.36%)
Pleural effusion	6 (12.77%)
Plasma	47 (100%)

### Platform comparison of EGFR mutation detection in tumor tissue

**Figure 2:**
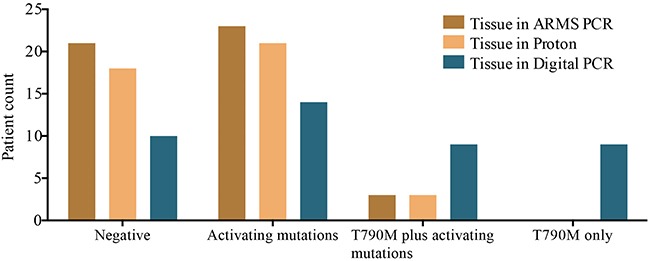
Classification of patients by EGFR mutation status identified by three platforms The x-axis is classified into four groups: no mutations detected, either L858R or exon 19 deletions or double mutants, referred to as activating mutations, T790M plus activating mutations, and T790M mutation only. The y-axis shows patient counts according to different mutation patterns detected in the three platforms.

We first compared the performance of three platforms in their abilities to detect mutations in tumor tissue. Using ARMS PCR results as the gold standard, high sensitivity was observed for both Proton and digital PCR, which had sensitivities of 100.00% (25/25) and 96.00% (24/25), respectively. However, digital PCR failed to detect one exon 19 deletion (Table 2). The mutation patterns of tumor tissue classified by the three platforms were different. ARMS PCR showed that 48.94% (23/47) of the patients harbored activating mutations, 6.38% (3/47) had a co-occurrence of activating mutations and the T790M mutation, and zero had only the T790M mutation. For the Proton platform, 50.00% (21/42) of the patients harbored activating mutations, 7.14% (3/42) had the T790M mutation plus activating mutations, and zero had only the T790M mutation. However, digital PCR detected activating mutations in 33.33% (14/42) of the patients, a co-occurrence of activating mutations and the T790M mutation in 21.43% (9/42), and only the T790M mutation in 21.43% (9/42). These results indicated that digital PCR could identify more gene mutations and thus provides more information for clinical decision-making (Figure 2; Supplementary Table 2).

**Table 2:** Performance of Proton and Digital PCR for detecting of EGFR mutation from tumor tissue DNA compared with ARMS PCR

EGFR mutation	ARMS	Proton		Performance of Proton			Digital PCR		Performance of Digital PCR		
		Positive	Negative	Sensitivity (%)	Specificity (%)	Concordance (%)	Positive	Negative	Sensitivity (%)	Specificity (%)	Concordance (%)
L858R	Positive	9	0	100%	100%	100%	9	0	100%	96.97%	97.62%
	Negative	0	33				1	32			
19 DEL	Positive	14	0	100%	89.29%	92.86%	13	1	92.86%	89.29%	90.48%
	Negative	3	25				3	25			
T790M	Positive	2	0	100%	97.50%	97.62%	2	0	100%	60.00%	61.90%
	Negative	1	39				16	24			
Total*	Positive	25	0	100%	94.17%	96.83%	24	1	96.00%	80.20%	83.33%
	Negative	4	97				20	81			

*. Comparison of Proton/Digital PCR with ARMS PCR was based on 42 tumor biopsies all tested in three platforms. Each EGFR mutation type had 42 sites’ result, the sensitivity and specificity were counted take site as unit.

Since the digital PCR was shown to be discordant with AMRS PCR and Proton, we next compared the difference in EGFR mutation abundance between Proton and digital PCR. Compared with Proton, digital PCR had a higher sensitivity, with results of 100%, 94.12% and 100.00% for L858R, exon 19 deletions and T790M mutations, respectively (Table 3). Of the seventeen discordant mutations (Supplementary Table 3), one L858R positive mutation in digital PCR was determined as negative in Proton, and digital PCR missed an exon 19 deletion mutation that was identified by Proton with an abundance of 2.91%. The other fifteen T790M mutations were identified by digital PCR only and had mutation frequencies that ranged from 0.10% to 0.32%. Overall, digital PCR was more sensitive than ARMS PCR and Proton in detecting EGFR mutations, especially in the detection of low abundance T790M mutations.

**Table 3:** Concordance between Digital PCR and Proton for detection of EGFR mutations from tumor tissue DNA

Mutation type	Proton	Digital PCR		Performance of Digital PCR		
		Positive	Negative	Sensitivity (%)	Specificity (%)	Concordance (%)
L858R	Positive	9	0	100%	96.97%	97.62%
	Negative	1	32			
19 DEL	Positive	16	1	94.12%	100%	97.62%
	Negative	0	25			
T790M	Positive	3	0	100%	61.54%	64.29%
	Negative	15	24			
Total	Positive	28	1	96.55%	83.51%	86.51%
	Negative	16	81			

The comparison was limited in a total of 42 tumor tissue samples detected using Proton and Digital PCR.

### Concordance of EGFR mutation detection in plasma and tissue

Due to having an insufficient amount of ctDNA to analyze all of the gene mutations (L858R, T790M and exon 19 deletion mutations) in each patient, we performed a total of 111 EGFR mutation (L858R: 35; T790M: 39; exon 19 deletions: 37) in 47 plasma samples by digital PCR. Compared with ARMS PCR in the detection of mutations in tumor tissue, 91.89% (102/111) of the sites were fully concordant, with twenty-three being positively concordant and seventy-nine being negatively concordant. The remaining 8.11% (9/111) of the sites were discordant, with five of them being detected in only tumor tissue and four being detected in only plasma ctDNA. Importantly, three of the four positive sites that were detected in only the ctDNA were validated in Proton or digital PCR tumor tissue. Benchmarking against the ARMS PCR tumor tissue results, which was used as the gold standard, the sensitivity and concordance rates for plasma mutation detection by digital PCR were 77.78% and 94.29%, respectively, for the L858R mutation, 82.35% and 83.38%, respectively, for the exon 19 deletion, and 100.00% and 97.44%, respectively, for the T790M mutation. The overall sensitivity, specificity and concordance were 82.14%, 95.18% and 91.89%, respectively (Table 4). ctDNA detection using digital PCR showed a high concordance with ARMS PCR.

**Table 4:** Concordance of EGFR mutation between tumor tissue detected using ARMS PCR and plasma detected using digital PCR

Mutation type	Tumor tissue	Plasma		Performance of plasma		
		Positive	Negative	Sensitivity (%)	Specificity (%)	Concordance (%)
L858R	Positive	7	2	77.78%	100.00%	94.29%
	Negative	0	26			
19 del	Positive	14	3	82.35%	85.00%	83.38%
	Negative	3	17			
T790M	Positive	2	0	100.00%	97.30%	97.44%
	Negative	1	36			
Total*	Positive	23	5	82.14%	95.18%	91.89%
	Negative	4	79			

*. Total was counted by three EGFR mutation types.

**Figure 3:**
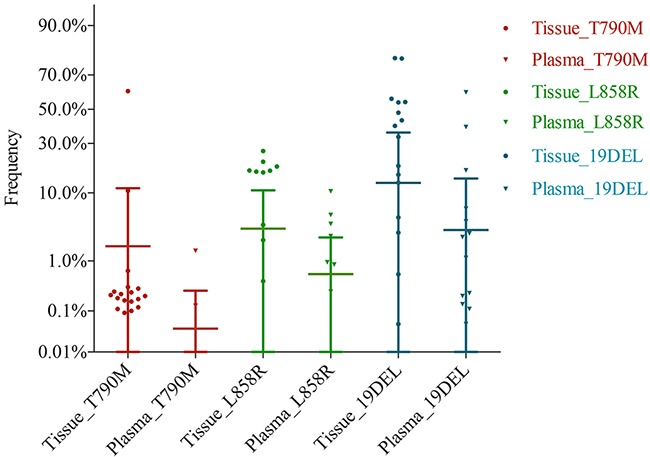
EGFR mutation frequency distribution of paired tumor tissue and plasma gDNA and ctDNA were detected in the digital PCR platform, and the frequency of tumor tissue is shown as a dot symbol, while the frequency of plasma is shown as a triangle symbol. Mutation types are shown by different colors. The frequency of plasma was lower than that of tumor tissue, and plasma did not detect a few positive sites in tumor tissue.

**Figure 4:**
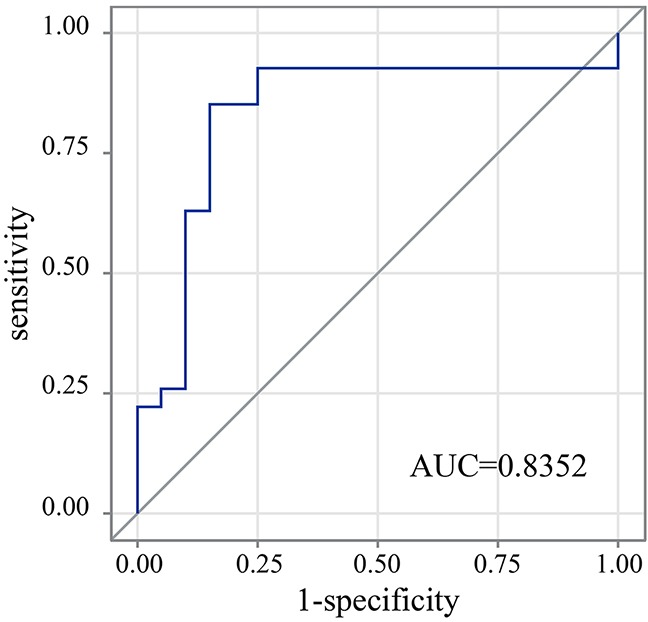
ROC analysis of plasma sample detection from tumor tissue mutation positive samples The AUC value is 0.8352, which is significant, with a P value of 0.0091. The cut-off value = max (sensitivity + specificity −1). The figure also shows a maximum sensitivity and specificity of 85.19% and 85.00%, respectively.

To further evaluate the detection performance of digital PCR for ctDNA and analyze the relationship between mutation abundance in tumor tissue and mutation abundance in ctDNA, we compared the consistence between tumor tissue and ctDNA in the digital PCR platform. To eliminate the confusion from sample types, ctDNA mutation detection was compared with genomic DNA (gDNA) detection in tumor tissue and pleural effusion separately. ctDNA identified all of the mutation types detected in pleural effusion. However, the detection rate varied for different mutation abundance levels between tumor tissue gDNA and ctDNA. The detection rates of plasma, compared to paired tumor tissue, were 3.03% when tumor tissue abundance was below 0.1%, 66.67% when tumor tissue abundance was between 0.5% and 1%, 50.00% when tumor tissue abundance was between 1% and 5%, and 87.50% when tumor tissue abundance was above 5% (Table 5). Of the twenty-two discordant mutations (Supplementary Table 4), fourteen of the T790M mutations that were negative in ctDNA were positive in tumor tissue, with a frequency below 0.32%. The others were three L858R and five exon 19 deletion mutations. The mutation abundance distribution of tumor tissue was higher than that of the paired ctDNA, and it appeared that the use of ctDNA could not identify mutations when the mutation abundance was very low in the paired tumor tissue (Figure 3). We further compared the relationship between the mutations identified in both tumor tissue and plasma. The Wilcoxon matched-pairs signed-rank test showed that there was a statistically significant difference between the abundance distributions in the two sample types (P<0.0001), and the Spearman’s rank correlation coefficient showed them to have a positive correlation, with an R^2^ value of 0.6274 (P< 0.0001). We further evaluated the detection sensitivity and specificity of plasma mutations obtained using digital PCR by comparing them with the mutation abundance in tumor tissue or pleural effusion samples. We performed receiver operator characteristic curve (ROC) analysis to evaluate the relationship between the mutation abundance in tumor tissue and the corresponding mutation status in plasma and achieved an area under the curve (AUC) value of 0.8352 (Z=0.1408, P=0.0091). With the optimal sensitivity and specificity being 85.19% and 85.00%, respectively, the cut-off value for the mutation abundance in tumor tissue was determined to be 3.81% (Figure 4; Supplementary Table 5). The interpretation of this is that when the mutation abundance in tumor tissue is lower than 3.81%, it is difficult to detect the corresponding mutation in plasma.

**Table 5:** Detection rate of plasma according to tumor tissue abundance

Tumor tissue abundance	Plasma		Performance of Plasma
	Negative	Positive	Detection rate (%)
MAF<0.1%	64	2	3.03% (2/66)
0.1%<=MAF<0.5%	10	0	0.00% (0/10)
0.5%<=MAF<1%	1	2	66.67% (2/3)
1%<=MAF<5%	2	2	50.00% (2/4)
MAF>=5%	3	21	87.50% (21/24)

## DISCUSSION

In this study, we have demonstrated that digital PCR is a low detection limit method for detecting low abundance mutations in tumor tissue and ctDNA based on the following results: (1) digital PCR had a higher detection rate of EGFR mutations than ARMS PCR or Proton, (2) digital PCR outperformed Proton in the detection of low abundance mutations, and (3) ctDNA mutations detected by digital PCR had a high rate of concordance with tumor tissue mutations detected by ARMS PCR. We also have shown that the use of digital PCR can achieve a high sensitivity for identifying mutations in ctDNA when the mutation abundance in paired tumor tissue DNA is above 3.81%.

A high sensitivity method with a low detection limit may increase the mutation detection rate in patients with low abundance T790M mutation. In this study, the EGFR T790M mutation detection rate in tumor tissue obtained by digital PCR was 42.86% (18/42). This detection rate was lower than that of droplet digital PCR (ddPCR), which was 79.9% in tumor tissue, and our lower T790M detection rate might be partly due to the 30 ng DNA input, as ddPCR required at least 50-100 ng DNA input to ensure the sensitivity to detect a mutation abundance as low as 0.009% [19]. DNA input might influence mutation detection because decreasing the DNA input would lead to a declined sensitivity [20]. Unlike Proton, which has a low detection limit of 1% that is subject to background noise levels [21], digital PCR provides a linear response to the number of target DNA copies and thus allows the precise measurement of the target concentration. The DNA sample is partitioned in digital PCR so that individual DNA molecules within the sample are divided into many separate regions, and DNA molecules are then quantified by counting the regions containing positive reactions after PCR [22]. A previous study has demonstrated that patients who harbored low abundance of active EGFR mutation might benefit less from EGFR-TKI therapy [16], however, for the EGFR T790M mutation, the detection of low abundance may relate to clinical practice. As we know, EGFR T790M mutation was an independent predictor of decreased progression-free survival (PFS) in patients with NSCLC who received TKI treatment [23], pre-existing EGFR T790M clones may induce early EGFR T790M-acquired resistance [24]. Therefore, low limit detection may help identify patients with resistance mutation in advance so that they can obtain more opportunities to prevent or overcome resistance in the clinic.

Circulating tumor DNA can be used for mutation detection in clinical practice, tumor burden monitoring, and dynamic surveillance to overcome difficulties of re-biopsy and tumor heterogeneity. Several studies had confirmed that EGFR mutations in NSCLC could be detected in ctDNA, and a meta-analysis reported that ctDNA offered 62.00% sensitivity and 95.90% specificity compared with tumor tissue DNA among the Asian population [12]. In the current study, the sensitivity and specificity of ctDNA detected by digital PCR were 82.14% and 95.18%, respectively, compared with tumor tissue detected by ARMS PCR. The use of digital PCR facilitated the mutation detection rate in ctDNA. However, the ctDNA gained a sensitivity of 55.32% compared with the tumor tissue DNA in the digital PCR platform, the concordance rate between tumor tissue and ctDNA was very low at a low mutation level abundance in tumor tissue, and the mutation abundance distribution range in ctDNA (interquartile range of 1.50% to 6.37%) was lower than that in paired tumor tissue DNA (interquartile range of 19.34% to 51.35%). This general observed trend was consistent with other oncogenic advanced studies that focused on NSCLC patients with advanced disease [15], which suggests that the reason might be that the mutated allele was too low in some tumor tissues, and once released into plasma, ctDNA was further diluted and thus became undetectable. Next, we used the ROC to explore the relationship of mutation abundance between ctDNA and tumor tissue. This showed that with the cutoff of 3.81% mutation abundance in tumor tissue, mutation status in paired ctDNA could be identified with optimized sensitivity and specificity (85.19% and 85.00%, respectively). This result suggested that when the frequency in tumor tissue was higher than 3.81%, a higher possibility to identify EGFR mutation in plasma by digital PCR would exist; otherwise, it might become undetectable.

Understanding the detection limit of ctDNA may inform strategies for dynamic surveillance and targeted therapy. For example, mutations detected in ctDNA were related to mutations in tumor tissue with an abundance higher than 3.81%. Since a previous study reported that T790M abundance was 2.0% in a post-therapy sample taken after clinical relapse [25] and the risk of disease progression after EGFR-TKI therapy in NSCLC patients began to increase while the T790M mutation abundance was at 3.2% [26], we recommended that once mutations are detected in ctDNA, patients should consider taking the targeted therapy to gain the greatest benefit. However, for those negative for mutations in ctDNA, it is still unclear whether the undetected reason was due to no mutation or a low abundance of mutations. In this case, patients could further confirm their gene mutation status by tissue biopsy or long-term plasma monitoring. The current study has demonstrated that digital PCR outperformed in the detection of low abundance mutations, so we suggest that digital PCR could be used as a high-sensitivity platform to overcome the low detection rate of ctDNA compared with tumor tissue. Overall, the relationship of mutation abundance between tumor tissue and ctDNA needs further investigation, but lower detection limit methods may be useful for a more detailed assessment of mutant allelic levels and a potential correlation with clinical response.

## MATERIALS AND METHODS

### Ethical compliance

The patient information and clinical samples were obtained from the First Affiliated Hospital of SunYat-sen University. The sample collection and preparation protocols were approved by the SunYat-sen Hospital Ethics Committee. All of the patients provided written informed consent and consented to gene analysis prior to being included in this study.

### Patient enrollment and sample collection

Forty-seven patients from the First Affiliated Hospital of SunYat-sen University were enrolled based on the following inclusion criteria: 1) patients with a diagnosis of stage III or IV NSCLC, 2) patients with a histological type of adenocarcinoma, and 3) treatment naïve patients or those who experienced disease progression after first-generation EGFR-TKI therapy at least one week prior to enrolling in the study. Furthermore, tumor tissue/pleural effusion and plasma samples were collected from the patients under the following procedure: 1) either matched tissue and plasma samples were collected at the time of surgery or plasma samples were collected before tissue samples by no more than one week [27]; 2) surgical tumor tissue was fixed in 10% buffered formalin as quickly as possible after surgical removal, then embedded in paraffin blocks and sectioned; 3) to obtain enough genomic DNA yields, 4-8 pieces of paraffin sections (5-10 μm thickness, 1×1 cm^2^ surface area) and one additional H&E slide were required for marking for tumor enrichment by a pathologist, and then, dissection of tissue from unstained tissue was performed; 4) pleural effusion samples were collected simultaneously when tissue biopsies might not be sufficient for gene analysis; and 5) at least 80-100 ml from pleural effusion samples was required, and at least 10-15 ml blood was required for the extraction of a sufficient amount of ctDNA.

An ARMS PCR assay of tumor tissue samples from the forty-seven patients was performed using the AmoyDx EGFR Gene mutation Detection Kit (Amoy Diagnostics, Xiamen, China). Due to a lack of a sufficient amount of tumor samples, pleural effusion samples were collected from six patients. Genomic DNA from tumor tissue/pleural effusions was sequenced in Proton and digital PCR, and ctDNA from forty-seven plasma samples was analyzed in digital PCR only. Because of the lack of a sufficient amount of ctDNA, sites detected with a mutation in the corresponding tumor tissue samples were assessed with priority (see Supplementary Figure 1 for the overall study design).

### DNA extraction and quantification

All extractions were conducted according to the manufacturer’s instructions. gDNA from FFPE samples was extracted using a TIANamp FFPE DNA Kit (Tiangen, Beijing, China). Pleural effusions were centrifuged at 3000 rpm for 15 min at RT, and then, gDNA was extracted from them using a TIANamp Blood DNA kit (Tiangen, Beijing, China). Before ctDNA extraction, plasma was obtained by centrifuging blood at 2000 x g for 10 min at 4°C, and the plasma layer was centrifuged at 16000 x g for 10 min at 4°C in order to further remove cell debris. Then, ctDNA was extracted from approximately 5 ml plasma using a QIAamp Circulating Nucleic Acid Kit (Qiagen, Valencia, CA) and eluted in a final volume of 30 μl. The DNA concentration was measured by a Qubit dsDNA HS assay kit on the Quantus Fluorometer according to the manufacturing protocol (Thermo Fisher Scientific, Waltham, MA). The DNA quality (A260/280) was determined by a Nanodrop 2000 spectrophotometer (Thermo Fisher Scientific, Waltham, MA) and agarose gel. The DNA was stored at −80°C for future use.

### Library preparation and sequence data processing

A total of 10-20 ng gDNA was used as the template, according to different DNA qualities. DNA libraries were prepared using an Ion AmpliSeq Library Kit 2.0 and Ion AmpliSeq Cancer Hotspot Panel V2, and ligated adapters were prepared using an Ion Xpress Barcode Adapter 1-96 kit (Thermo Fisher Scientific, Waltham, MA) according to the manufacturer’s protocol. The library concentration was quantified using a Qubit dsDNA HS assay kit (Thermo Fisher Scientific, Waltham, MA) and the Agilent 2100 Bioanalyzer instrument. Based on the result, emulsion PCR and enrichment steps were prepared using an Ion OneTouch 2 System (Thermo Fisher Scientific, Waltham, MA). Then, the amplicon library was sequenced via an Ion Proton system with an Ion PI chip (Thermo Fisher Scientific, Waltham, MA).

Data analysis was performed based on the Torrent Suite Software version 4.4 (Thermo Fisher Scientific, Waltham, MA). The sequencing reads were aligned to the reference human genome sequence (hg19) using the Torrent Mapping Alignment Program (TMAP) with the default parameters. Quality control was performed following the manufacturer’s standard protocol, the average depth per sample required higher than 3000X, and the variant depth required higher than 4500X. However, to enhance EGFR exon 19 deletions, we added an additional Torrent Variant Caller (TVC) step with modified TMAP parameter (−-softclip-type 3, do not allow soft-clipping), hotspot VCF file and bed file of EGFR exon 19. For a mutation abundance below 1%, we used IGV software to make up for TVC being missing. Mutations were determined to be positive when IGV clearly presented an abundance higher than 1%.

### Detection of EGFR mutations by QuantStudio 3D Digital PCR

Digital PCR assays were conducted using a QuantStudio 3D Digital PCR system (Thermo Fisher Scientific, Waltham, MA). A total of 30 ng gDNA/ctDNA was amplified using Taqman SNP Genotyping Assays, in which the probes were chosen according to specific EGFR gene mutations (L858R, T790M and exon 19 deletions). Since three EGFR mutation types were analyzed in a single assay, samples without enough ctDNA (90 ng) would exhibit a positive mutation prior to the negative sites, according to the detection in paired tumor tissue, and when ctDNA was less than 30 ng, we did not perform the assay. PCR reagents were loaded with a QuantStudio 3D Digital PCR 20K Chip v2. The subsequent analysis and post-processing were performed with the QuantStudio 3D AnalysisSuite Software.

### Statistics

The statistical data were analyzed with SAS software ver. 9.2 (SAS Institute, Cary, NC). The Wilcoxon matched-pairs signed-rank test was used to compare the mutation abundance detection in tumor tissue and plasma using the digital PCR platform. Spearman’s rank correlation coefficient was used to analyze the correlation between tumor frequency and plasma mutation abundance. ROC analysis was used to evaluate the detection sensitivity, specificity and cut-off value of tumor tissue mutation abundance. P<0.05 was considered statistically significant.

## SUPPLEMENTARY MATERIALS FIGURE AND TABLES







## Abbreviations

ctDNA: circulating tumor DNA
NSCLC: non-small cell lung cancer
EGFR: epidermal growth factor receptor
EGFR TKIs: EGFR tyrosine kinase inhibitors
NGS: next-generation sequencing
PCR: polymerase chain reaction
PD: progressive disease
ARMS: Scorpion amplification refractory mutation system
gDNA: genomic DNA
TMAP: Torrent Mapping Alignment Program
TVC: Torrent Variant Caller
ddPCR: droplet digital PCR
MAF: mutation allele frequency
FFPE: formalin-fixed paraffin-embedded.
