# Supplementary material for: Evaluation of digital PCR for detecting low-level EGFR mutations in advanced lung adenocarcinoma patients: a cross-platform comparison study
Source: Oncotarget. 2017 Jun 29;8(40):67810–20. doi: 10.18632/oncotarget.18866 (PMC5620214; doi:10.18632/oncotarget.18866)
Supplement: Supplementary file 1 [file oncotarget-08-67810-s001.pdf]

Evaluation of digital PCR for detecting low-level EGFR mutations in advanced lung adenocarcinoma patients: a cross-platform comparison study

SUPPLEMENTARY MATERIALS

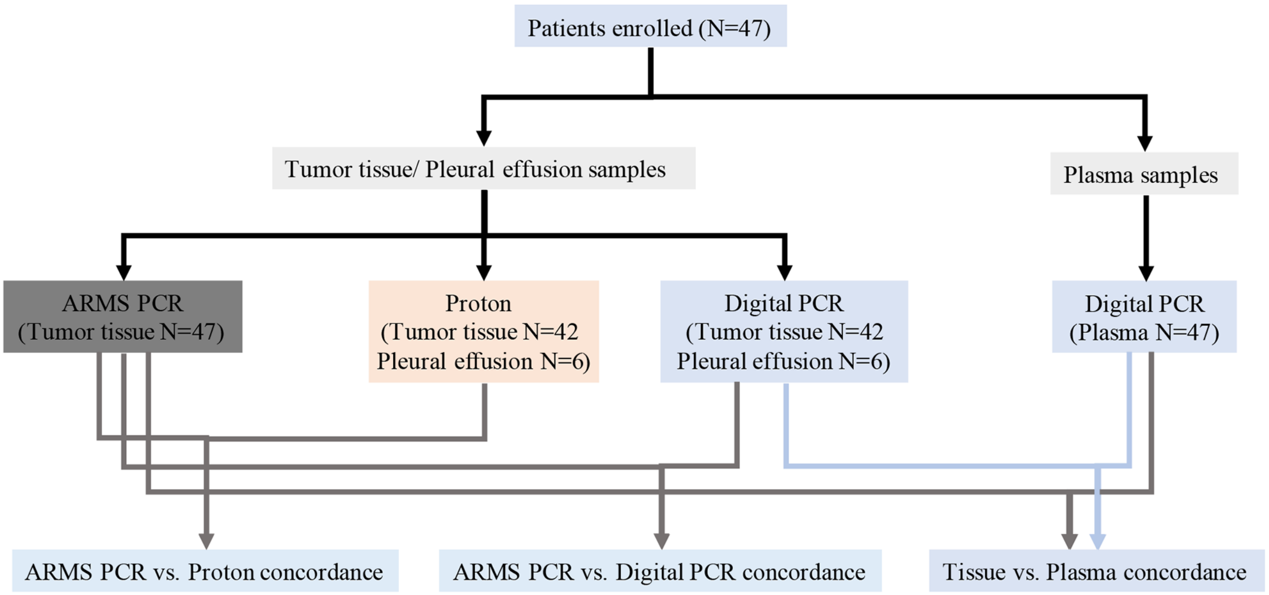

Supplementary Figure 1: Study design for the detection of EGFR mutations in matched tumor tissue and plasma specimens from NSCLC patients. Paired tumor tissue and plasma samples were collected from the enrolled NSCLC patients. Tumor tissue samples were detected using ARMS PCR, Proton, and digital PCR methods and platforms, while plasma samples were detected using digital PCR only.

Supplementary Table 1: Summary of EGFR status identified by ARMS PCR, Proton and digital PCR.

See Supplementary File 1

Supplementary Table 2: Classification of EGFR mutation status identified by three platforms

Mutation pattern	Tumor tissue					
	ARMS**	Percent	Proton***	Percent	Digital PCR***	Percent
No mutation detected	21	44.68%	18	42.86%	10	23.81%
Activating mutations*	23	48.94%	21	50.00%	14	33.33%
T790M plus activating mutations	3	6.38%	3	7.14%	9	21.43%
T790M only	0	0.00%	0	0.00%	9	21.43%

*. Patients harbored EGFR exon 19 deletion and/ or L858R mutations; **. There was a total of 47 tumor tissue samples detected using ARMS PCR; ***. There was a total of 42 tumor tissue samples detected using Proton and Digital PCR, other 5 were pleural effusion samples. As comparison limited in tumor tissue, we only calculated 42 tumor tissue.

Supplementary Table 3: Discordant results with Proton and digital PCR detection of the EGFR mutations from tumor tissue DNA

Sample ID	Gender	Stage	Gene	Mutation	ARMS tissue	Proton tissue		Digital PCR tissue	
					Positive/negative	Positive/negative	MAF	Positive/negative	MAF
P06	Male	IIIB	EGFR	T790M	N	N		P	0.16%
P12	Female	IV	EGFR	L858R	N	N	0.36%	P	0.42%
P13	Male	IV	EGFR	T790M	N	N		P	0.10%
P18	Female	IV	EGFR	T790M	N	N		P	0.11%
P20	Female	IV	EGFR	T790M	N	N		P	0.21%
P22	Male	IV	EGFR	T790M	N	N	0.22%	P	0.10%
P23	Male	IV	EGFR	T790M	N	N	0.11%	P	0.22%
P27	Male	IV	EGFR	T790M	N	N		P	0.12%
P33	Female	IV	EGFR	T790M	N	N		P	0.32%
P34	Male	IV	EGFR	T790M	N	N		P	0.30%
P35	Female	IV	EGFR	T790M	N	N		P	0.25%
P36	Female	IV	EGFR	T790M	N	N		P	0.17%
P37	Male	IV	EGFR	T790M	N	N		P	0.19%
P38	Female	IV	EGFR	T790M	N	N		P	0.26%
P40	Male	IV	EGFR	T790M	N	N		P	0.18%
P40	Male	IV	EGFR	19del	P	P	2.91%	N	
P42	Female	IV	EGFR	T790M	N	N		P	0.23%

MAF., mutation allelic abundance.

Supplementary Table 4: Discordant EGFR mutations of tumor tissue and plasma

Sample ID	Gene	Mutation	ARMS tissue	Proton tissue		Digital PCR tissue		Digital PCR plasma	
			Positive/negative	Positive/ negative	MAF	Positive/negative	MAF	Positive/ negative	MAF
P06	EGFR	T790M	N	N		P	0.16%	N	
P08	EGFR	L858R	P	P	21.72%	P	16.91%	N	
P10	EGFR	L858R	P	P	1.96%	P	2.23%	N	
P12	EGFR	L858R	N	N	0.36%	P	0.42%	N	
P13	EGFR	T790M	N	N		P	0.10%	N	
P18	EGFR	T790M	N	N		P	0.11%	N	
P20	EGFR	T790M	N	N		P	0.21%	N	
P22	EGFR	T790M	N	N	0.22%	P	0.10%	N	
P23	EGFR	T790M	N	N	0.11%	P	0.22%	N	
P28	EGFR	19del	N	N		N		P	2.49%
P31	EGFR	19del	P	P	55.71%	P	54.38%	N	
P33	EGFR	T790M	N	N		P	0.32%	N	
P34	EGFR	T790M	N	N		P	0.30%	N	
P34	EGFR	19del	P	P	48.91%	P	47.82%	N	
P35	EGFR	T790M	N	N		P	0.25%	N	
P36	EGFR	T790M	N	N		P	0.17%	N	
P37	EGFR	T790M	N	N		P	0.19%	N	
P37	EGFR	19del	N	P	1.22%	P	0.57%	N	
P38	EGFR	T790M	N	N		P	0.26%	N	
P38	EGFR	19del	N	P	2.40%	P	2.91%	N	
P40	EGFR	T790M	N	N		P	0.18%	N	
P42	EGFR	T790M	N	N		P	0.23%	N	

MAF., mutation allelic abundance.

Supplementary Table 5: Cut-off value of ROC curve

See Supplementary File 2
