# Supplementary material for: Evaluation of digital PCR for detecting low-level EGFR mutations in advanced lung adenocarcinoma patients: a cross-platform comparison study
Source: Oncotarget. 2017 Jun 29;8(40):67810–20. doi: 10.18632/oncotarget.18866 (PMC5620214; doi:10.18632/oncotarget.18866)
Supplement: Supplementary file 2 [file oncotarget-08-67810-s002.docx]

**Supplementary Table 1: Summary of EGFR status identified by ARMS PCR, Proton and digital PCR**

| **Sample ID** | **Gender** | **Stage** | **Gene** | **Mutation** | **ARMS tissue** | **Proton tissue** | | **Digital PCR tissue** | | **Digital PCR plasma** | |
| --- | --- | --- | --- | --- | --- | --- | --- | --- | --- | --- | --- |
|  |  |  |  |  | **Positive/negative** | **Positive/negative** | **MAF** | **Positive/negative** | **MAF** | **Positive/negative** | **MAF** |
| P01 | Male | IV | EGFR | L858R | N | N |  | N |  | N |  |
|  |  |  |  | T790M | N | N |  | N |  | N |  |
|  |  |  |  | 19 DEL | N | N |  | N |  | N |  |
| P02 | Male | IV | EGFR | L858R | N | N |  | N |  | N |  |
|  |  |  |  | T790M | N | N |  | N |  | N |  |
|  |  |  |  | 19 DEL | N | N |  | N |  | N |  |
| P03 | Male | IV | EGFR | L858R | N | N |  | N |  | NA |  |
|  |  |  |  | T790M | N | N |  | N |  | N |  |
|  |  |  |  | 19 DEL | P | P | 19.39% | P | 15.77% | P | 0.10% |
| P04 | Male | IV | EGFR | L858R | N | N |  | N |  | N |  |
|  |  |  |  | T790M | N | N |  | N |  | N |  |
|  |  |  |  | 19 DEL | N | N |  | N |  | N |  |
| P05 | Male | IIIA | EGFR | L858R | N | N |  | N |  | N |  |
|  |  |  |  | T790M | N | N |  | N |  | N |  |
|  |  |  |  | 19 DEL | P | P | 14.06% | P | 12.97% | P | 0.24% |
| P06 | Male | IIIB | EGFR | L858R | N | N |  | N |  | N |  |
|  |  |  |  | T790M | N | N |  | P | 0.16% | N |  |
|  |  |  |  | 19 DEL | N | N |  | N |  | N |  |
| P07 | Female | IV | EGFR | L858R | P | P | 25.79% | P | 26.02% | P | 0.26% |
|  |  |  |  | T790M | N | N |  | N |  | N |  |
|  |  |  |  | 19 DEL | N | N |  | N |  | N |  |
| P08 | Female | IIIA | EGFR | L858R | P | P | 21.72% | P | 16.91% | N |  |
|  |  |  |  | T790M | N | N |  | N |  | NA |  |
|  |  |  |  | 19 DEL | N | N |  | N |  | N |  |
| P09 | Male | IV | EGFR | L858R | N | N |  | N |  | NA |  |
|  |  |  |  | T790M | N | N |  | N |  | NA |  |
|  |  |  |  | 19 DEL | N | N |  | N |  | N |  |
| P10 | Female | IV | EGFR | L858R | P | P | 1.96% | P | 2.23% | N |  |
|  |  |  |  | T790M | N | P | 0.50% | P | 0.66% | P | 0.13% |
|  |  |  |  | 19 DEL | N | N |  | N |  | NA |  |
| P11 | Male | IV | EGFR | L858R | P | P | 18.09% | P | 16.61% | P | 10.41% |
|  |  |  |  | T790M | N | N |  | N |  | N |  |
|  |  |  |  | 19 DEL | N | N |  | N |  | N |  |
| P12 | Female | IV | EGFR | L858R | N | N | 0.36% | P | 0.42% | N |  |
|  |  |  |  | T790M | N | N |  | N |  | NA |  |
|  |  |  |  | 19 DEL | P | P | 40.80% | P | 39.91% | P | 39.19% |
| P13 | Male | IV | EGFR | L858R | N | N | 0.18% | N |  | N |  |
|  |  |  |  | T790M | N | N |  | P | 0.10% | N |  |
|  |  |  |  | 19 DEL | N | N |  | N |  | N |  |
| P14 | Male | IV | EGFR | L858R | N | N |  | N |  | NA |  |
|  |  |  |  | T790M | P | P | 10.80% | P | 10.58% | NA |  |
|  |  |  |  | 19 DEL | P | P | 46.12% | P | 43.17% | P | 0.21% |
| P15 | Female | IIIA | EGFR | L858R | N | N |  | N |  | NA |  |
|  |  |  |  | T790M | N | N |  | N |  | N |  |
|  |  |  |  | 19 DEL | N | N |  | N |  | NA |  |
| P16 | Male | IIIB | EGFR | L858R | N | N |  | N |  | NA |  |
|  |  |  |  | T790M | N | N |  | N |  | N |  |
|  |  |  |  | 19 DEL | N | N |  | N |  | NA |  |
| P17 | Female | IV | EGFR | L858R | P | P | 19.44% | P | 17.38% | P | 0.86% |
|  |  |  |  | T790M | N | N |  | N |  | NA |  |
|  |  |  |  | 19 DEL | N | N |  | N |  | NA |  |
| P18 | Female | IV | EGFR | L858R | N | N |  | N |  | NA |  |
|  |  |  |  | T790M | N | N |  | P | 0.11% | N |  |
|  |  |  |  | 19 DEL | N | N |  | N |  | NA |  |
| P19 | Female | IIIA | EGFR | L858R | P | P | 22.27% | P | 21.02% | P | 0.95% |
|  |  |  |  | T790M | N | N |  | N |  | NA |  |
|  |  |  |  | 19 DEL | N | P | 1.02% | P | 0.10% | P | 0.11% |
| P20 | Female | IIIA | EGFR | L858R | N | N |  | N |  | NA |  |
|  |  |  |  | T790M | N | N |  | P | 0.21% | N |  |
|  |  |  |  | 19 DEL | N | N |  | N |  | NA |  |
| P21 | Male | IV | EGFR | L858R | N | N |  | N |  | NA |  |
|  |  |  |  | T790M | N | N |  | N |  | NA |  |
|  |  |  |  | 19 DEL | P | P | 39.50% | P | 33.51% | P | 2.85% |
| P22 | Male | IV | EGFR | L858R | N | N |  | N |  | NA |  |
|  |  |  |  | T790M | N | N | 0.22% | P | 0.10% | N |  |
|  |  |  |  | 19 DEL | N | N |  | N |  | NA |  |
| P23 | Male | IV | EGFR | L858R | P | P | 19.74% | P | 19.01% | P | 2.58% |
|  |  |  |  | T790M | N | N | 0.11% | P | 0.22% | N |  |
|  |  |  |  | 19 DEL | N | N |  | N |  | NA |  |
| P24 | Male | IIIB | EGFR | L858R | N | N |  | N |  | NA |  |
|  |  |  |  | T790M | N | N |  | N |  | N |  |
|  |  |  |  | 19 DEL | N | N |  | N |  | NA |  |
| P25 | Female | IV | EGFR | L858R | N | N |  | N |  | NA |  |
|  |  |  |  | T790M | N | N |  | N |  | N |  |
|  |  |  |  | 19 DEL | N | N |  | N |  | NA |  |
| P26 | Female | IV | EGFR | L858R | N | N |  | N |  | NA |  |
|  |  |  |  | T790M | N | N |  | N |  | NA |  |
|  |  |  |  | 19 DEL | P | P | 22.54% | P | 19.18% | P | 6.37% |
| P27 | Female | IV | EGFR | L858R | N | N |  | N |  | NA |  |
|  |  |  |  | T790M | N | N |  | P | 0.12% | NA |  |
|  |  |  |  | 19 DEL | P | P | 58.20% | P | 54.08% | P | 17.40% |
| P28 | Male | IIIA | EGFR | L858R | N | N |  | N |  | N |  |
|  |  |  |  | T790M | N | N |  | N |  | N |  |
|  |  |  |  | 19 DEL | N | N |  | N |  | P | 2.49% |
| P29 | Female | IV | EGFR | L858R | N | N |  | N |  | N |  |
|  |  |  |  | T790M | N | N |  | N |  | N |  |
|  |  |  |  | 19 DEL | N | N |  | N |  | N |  |
| P30 | Male | IV | EGFR | L858R | N | N |  | N |  | N |  |
|  |  |  |  | T790M | N | N |  | N |  | N |  |
|  |  |  |  | 19 DEL | P | P | 76.79% | P | 78.26% | P | 1.13% |
| P31 | Female | IV | EGFR | L858R | N | N |  | N |  | N |  |
|  |  |  |  | T790M | N | N |  | N |  | N |  |
|  |  |  |  | 19 DEL | P | P | 55.71% | P | 54.38% | N |  |
| P32 | Male | IV | EGFR | L858R | N | N |  | N |  | N |  |
|  |  |  |  | T790M | N | N |  | N |  | N |  |
|  |  |  |  | 19 DEL | P | P | 3.09% | P | 4.84% | P | 0.14% |
| P33 | Male | IV | EGFR | L858R | N | N |  | N |  | N |  |
|  |  |  |  | T790M | N | N |  | P | 0.32% | N |  |
|  |  |  |  | 19 DEL | N | N |  | N |  | NA |  |
| P34 | Female | IV | EGFR | L858R | N | N |  | N |  | N |  |
|  |  |  |  | T790M | N | N |  | P | 0.30% | N |  |
|  |  |  |  | 19 DEL | P | P | 48.91% | P | 47.82% | N |  |
| P35 | Female | IV | EGFR | L858R | N | N |  | N |  | N |  |
|  |  |  |  | T790M | N | N |  | P | 0.25% | N |  |
|  |  |  |  | 19 DEL | N | N |  | N |  | N |  |
| P36 | Male | IIB | EGFR | L858R | N | N |  | N |  | N |  |
|  |  |  |  | T790M | N | N |  | P | 0.17% | N |  |
|  |  |  |  | 19 DEL | N | N |  | N |  | N |  |
| P37 | Female | IV | EGFR | L858R | N | N |  | N |  | N |  |
|  |  |  |  | T790M | N | N |  | P | 0.19% | N |  |
|  |  |  |  | 19 DEL | N | P | 1.22% | P | 0.57% | N |  |
| P38 | Male | IV | EGFR | L858R | P | P | 16.90% | P | 17.27% | P | 3.92% |
|  |  |  |  | T790M | N | N |  | P | 0.26% | N |  |
|  |  |  |  | 19 DEL | N | P | 2.40% | P | 2.91% | N |  |
| P39 | Male | IV | EGFR | L858R | P | P | 2.20% | P | 3.81% | P | 5.24% |
|  |  |  |  | T790M | N | N |  | N |  | N |  |
|  |  |  |  | 19 DEL | N | N |  | N |  | N |  |
| P40 | Male | IV | EGFR | L858R | N | N |  | N |  | N |  |
|  |  |  |  | T790M | N | N |  | P | 0.18% | N |  |
|  |  |  |  | 19 DEL | P | P | 2.91% | N |  | N |  |
| P41 | Female | IV | EGFR | L858R | N | N |  | N |  | N |  |
|  |  |  |  | T790M | P | P | 69.45% | P | 60.84% | P | 1.50% |
|  |  |  |  | 19 DEL | P | P | 79.07% | P | 78.15% | P | 4.26% |
| P42 (Tumor tissue) | Female | IV | EGFR | L858R | N | N |  | N |  | N |  |
|  |  |  |  | T790M | N | N |  | P | 0.23% | N |  |
|  |  |  |  | 19 DEL | P | P | 53.29% | P | 56.31% | P | 60.18% |
| P42 (PE) | Female | IV | EGFR | L858R | N | N |  | N |  | N |  |
|  |  |  |  | T790M | N | N |  | N |  | N |  |
|  |  |  |  | 19 DEL | P | P | 16.73% | P | 83.78% | P | 60.18% |
| P43 (PE) | Male | IV | EGFR | L858R | N | N |  | N |  | N |  |
|  |  |  |  | T790M | N | N |  | N |  | N |  |
|  |  |  |  | 19 DEL | N | P | 0.49% | P | 0.75% | P | 11.51% |
| P44 (PE) | Male | IV | EGFR | L858R | N | N |  | N |  | N |  |
|  |  |  |  | T790M | P | P | 9.02% | P | 8.01% | P | 0.27% |
|  |  |  |  | 19 DEL | P | P | 58.26% | P | 56.64% | P | 0.44% |
| P45 (PE) | Female | IV | EGFR | L858R | N | N |  | N |  | N |  |
|  |  |  |  | T790M | N | N |  | N |  | N |  |
|  |  |  |  | 19 DEL | N | N |  | N |  | N |  |
| P46 (PE) | Female | IV | EGFR | L858R | N | N |  | N |  | N |  |
|  |  |  |  | T790M | N | N |  | N |  | N |  |
|  |  |  |  | 19 DEL | P | P | 21.94% | P | 19.50% | P | 0.21% |
| P47 (PE) | Male | IIIB | EGFR | L858R | N | N |  | N |  | N |  |
|  |  |  |  | T790M | N | N |  | N |  | N |  |
|  |  |  |  | 19 DEL | N | N |  | N |  | N |  |

NA, not available; MAF., mutation allelic abundance; Pleural effusion samples were collected from six patients to detect EGFR mutations in Proton and digital PCR in comparison with plasma samples.
