# Supplementary material for: Evaluation of digital PCR for detecting low-level EGFR mutations in advanced lung adenocarcinoma patients: a cross-platform comparison study
Source: Oncotarget. 2017 Jun 29;8(40):67810–20. doi: 10.18632/oncotarget.18866 (PMC5620214; doi:10.18632/oncotarget.18866)
Supplement: Supplementary file 3 [file oncotarget-08-67810-s003.docx]

**Supplementary Table 5: Cut-off value of ROC curve**

| **Obs** | **Tumor tissue MAF** | **Plasma EGFR mutation status**  **(Positive/Negative)** | **Sensitivity** | **Specificity** | **(Sensitivity + Specificity)-1** |
| --- | --- | --- | --- | --- | --- |
| 1 | 0.8378 | P | 0.0370 | 1.0000 | 0.0370 |
| 2 | 0.7826 | P | 0.0741 | 1.0000 | 0.0741 |
| 3 | 0.7815 | P | 0.1111 | 1.0000 | 0.1111 |
| 4 | 0.6084 | P | 0.1482 | 1.0000 | 0.1482 |
| 5 | 0.5664 | P | 0.1852 | 1.0000 | 0.1852 |
| 6 | 0.5631 | P | 0.2222 | 1.0000 | 0.2222 |
| 7 | 0.5438 | N | 0.2222 | 0.9500 | 0.1722 |
| 8 | 0.5408 | P | 0.2593 | 0.9500 | 0.2093 |
| 9 | 0.4782 | N | 0.2593 | 0.9000 | 0.1593 |
| 10 | 0.4317 | P | 0.2963 | 0.9000 | 0.1963 |
| 11 | 0.3991 | P | 0.3333 | 0.9000 | 0.2333 |
| 12 | 0.3351 | P | 0.3704 | 0.9000 | 0.2704 |
| 13 | 0.2602 | P | 0.4074 | 0.9000 | 0.3074 |
| 14 | 0.2102 | P | 0.4444 | 0.9000 | 0.3444 |
| 15 | 0.195 | P | 0.4815 | 0.9000 | 0.3815 |
| 16 | 0.1918 | P | 0.5185 | 0.9000 | 0.4185 |
| 17 | 0.1901 | P | 0.5556 | 0.9000 | 0.4556 |
| 18 | 0.1738 | P | 0.5926 | 0.9000 | 0.4926 |
| 19 | 0.1727 | P | 0.6296 | 0.9000 | 0.5296 |
| 20 | 0.1691 | N | 0.6296 | 0.8500 | 0.4796 |
| 21 | 0.1661 | P | 0.6667 | 0.8500 | 0.5167 |
| 22 | 0.1577 | P | 0.7037 | 0.8500 | 0.5537 |
| 23 | 0.1297 | P | 0.7407 | 0.8500 | 0.5907 |
| 24 | 0.0801 | P | 0.7778 | 0.8500 | 0.6278 |
| 25 | 0.0484 | P | 0.8148 | 0.8500 | 0.6648 |
| 26 | 0.0381 | P | 0.8519 | 0.8500 | 0.7019 |
| 27 | 0.0291 | N | 0.8519 | 0.8000 | 0.6519 |
| 28 | 0.0223 | N | 0.8519 | 0.7500 | 0.6019 |
| 29 | 0.0075 | P | 0.8889 | 0.7500 | 0.6389 |
| 30 | 0.0066 | P | 0.9259 | 0.7500 | 0.6759 |
| 31 | 0.0057 | N | 0.9259 | 0.7000 | 0.6259 |
| 32 | 0.0042 | N | 0.9259 | 0.6500 | 0.5759 |
| 33 | 0.0032 | N | 0.9259 | 0.6000 | 0.5259 |
| 34 | 0.003 | N | 0.9259 | 0.5500 | 0.4759 |
| 35 | 0.0026 | N | 0.9259 | 0.5000 | 0.4259 |
| 36 | 0.0025 | N | 0.9259 | 0.4500 | 0.3759 |
| 37 | 0.0023 | N | 0.9259 | 0.4000 | 0.3259 |
| 38 | 0.0022 | N | 0.9259 | 0.3500 | 0.2759 |
| 39 | 0.0021 | N | 0.9259 | 0.3000 | 0.2259 |
| 40 | 0.0019 | N | 0.9259 | 0.2500 | 0.1759 |
| 41 | 0.0018 | N | 0.9259 | 0.2000 | 0.1259 |
| 42 | 0.0017 | N | 0.9259 | 0.1500 | 0.0759 |
| 43 | 0.0016 | N | 0.9259 | 0.1000 | 0.0259 |
| 44 | 0.0011 | N | 0.9259 | 0.0500 | -0.0241 |
| 45 | 0.0009 | N | 0.9259 | 0.0000 | -0.0741 |
| 46 | 0.0005 | P | 0.9630 | 0.0000 | -0.0370 |
| 47 | 0 | P | 1.0000 | 0.0000 | 0.0000 |
